# EANM guideline on quality risk management for radiopharmaceuticals

**DOI:** 10.1007/s00259-022-05738-4

**Published:** 2022-04-07

**Authors:** Nic Gillings, Olaug Hjelstuen, Martin Behe, Clemens Decristoforo, Philip H. Elsinga, Valentina Ferrari, Oliver C. Kiss, Petra Kolenc, Jacek Koziorowski, Peter Laverman, Thomas L. Mindt, Meltem Ocak, Marianne Patt, Sergio Todde, Almut Walte

**Affiliations:** 1 Department of Clinical Physiology and Nuclear Medicine, Copenhagen University Hospital Rigshospitalet, Copenhagen, Denmark; 2 GE Healthcare, Pharmaceutical Diagnostics, Oslo, Norway; 3 Center for Radiopharmaceutical Sciences, Paul Scherrer Institute, Villigen, Switzerland; 4 Department of Nuclear Medicine, Medical University Innsbruck, Innsbruck, Austria; 5 Department of Nuclear Medicine and Molecular Imaging, University Medical Center Groningen, Groningen, The Netherlands; 6 MSD Animal Health, Milton Keynes, UK; 7 Department of Radiopharmaceuticals Production, Institute for Radiopharmaceutical Cancer Research, Helmholtz-Zentrum Dresden-Rossendorf, Dresden, Germany; 8 Department of Nuclear Medicine and Faculty of Pharmacy, University Medical Centre Ljubljana and University of Ljubljana, Ljubljana, Slovenia; 9 Independent Consultant, Linköping, Sweden; 10 Department of Medical Imaging, Nuclear Medicine, Radboud University Medical Center Nijmegen, Nijmegen, The Netherlands; 11 Ludwig Boltzmann Institute Applied Diagnostics, Vienna, Austria; 12 Department of Pharmaceutical Technology, Istanbul University, Istanbul, Turkey; 13 Department for Nuclear Medicine, University Hospital Leipzig, Leipzig, Germany; 14 Tecnomed Foundation, University of Milano-Bicocca, Monza, Italy; 15 Department of Nuclear Medicine, Hannover Medical School, Hanover, Germany

**Keywords:** Risk assessment, Radiopharmaceuticals, Quality assurance

## Abstract

This document is intended as a supplement to the EANM “Guidelines on current Good Radiopharmacy Practice (cGRPP)” issued by the Radiopharmacy Committee of the EANM (Gillings et al. in EJNMMI Radiopharm Chem. 6:8, 2021). The aim of the EANM Radiopharmacy Committee is to provide a document that describes how to manage risks associated with small-scale “in-house” preparation of radiopharmaceuticals, not intended for commercial purposes or distribution.

## Preamble

The European Association of Nuclear Medicine (EANM) is a professional non-profit medical association that facilitates communication worldwide among individuals pursuing clinical and research excellence in nuclear medicine. The EANM was founded in 1985. This guideline has been written by members of the EANM Radiopharmacy Committee and is intended to assist professionals in the risk assessments regarding the small-scale “in-house” preparation of radiopharmaceuticals.

## Background

GMP Part I [1] and Part II [2] state that, to ensure that medicinal products are suitable for their intended use, comply with regulatory requirements and maintain product quality, safety and efficacy, the quality system must incorporate quality risk management (QRM). This highlights the importance of integrating a robust risk assessment process within the quality system.

GMP Part III [3] contains an entire section on QRM, risk management methods and tools and potential application for QRM. Its purpose is to provide guidance on more effective and consistent risk-based decisions and on some of the most frequently used risk assessment tools. The section also clarifies that it is not always appropriate to use recognised tools only, and the use of informal risk management processes can be considered acceptable.

Relevant references to the need to apply risk management and risk assessment are also included in various EU-GMP Annexes, such as Annexes 1, 3, 11, 13 and 15 [4–8]. The same principles are further stressed in other documents which, although not legally binding, nonetheless may provide useful guidance in focusing on specific aspects and related risks for radiopharmaceuticals. An example of such a document is the European Pharmacopoeia general chapter 5.19 on “Extemporaneous preparation of radiopharmaceuticals” [9], which is specifically dedicated to the preparation of small-scale, on-site preparation of radiopharmaceuticals and where risk assessment is frequently mentioned as a useful and necessary tool to determine the general level of risk and to make decisions on specific potential issues arising during the preparation of this class of radiopharmaceuticals.

Thus, it is clear why risk assessment principles have gained continuous and increasing attention over the last decades, and they are now considered an important aspect in the framework of the preparation of medicinal products. Thus, QRM principles should be used to assess and control risks associated with the preparation of any pharmaceuticals, including radiopharmaceuticals.

## Risk assessment in the preparation of radiopharmaceuticals

Radiopharmaceuticals are a special class of medicinal product, and, due to their inherent nature, it is commonly accepted that general rules and regulations applying to classic, non-radioactive pharmaceuticals need to be adapted in case of radiopharmaceutical preparations. Risk assessment should be viewed as a powerful tool that may help to take decisions and evaluate whether the above adaptations may be considered acceptable and ensure that they do not have an adverse impact on the patient.

There are specific characteristics of radiopharmaceutical preparations that might, in principle, decrease the risk compared with classical pharmaceutical preparations, such as:

- Radiopharmaceuticals are generally used within a few hours of their preparation (microbiological growth in case of contamination is negligible).
- Very small quantities of starting materials are normally used (e.g. in the mg scale). Masses associated with radiopharmaceuticals are often very low (micro-dosing concept), and thus toxicity concerns are often minimal.
- Toxicity is further reduced by the small number of times (often one time only) a radiopharmaceutical is typically administered during the whole life of a patient.
- Shelf lives of radiopharmaceuticals are often short or very short (from less than 1 h up to a few days), and risks related to long-term storage are negligible.
- Finally, “in-house” prepared radiopharmaceuticals are typically used internally, and there are no risks associated with a complex distribution chain.

## Quality risk management

QRM should enable the identification of risks and mitigate them with appropriate and robust controls, to ensure that product quality, safety and efficacy are maintained during the product life cycle.

The process of managing, especially the evaluation of the risks associated with the preparation of any pharmaceutical, including radiopharmaceuticals, should be based on scientific knowledge and is ultimately aimed at improving patient protection. A thorough knowledge of the products and critical processes are the basis for a solid and effective QRM program.

**Fig. 1:**
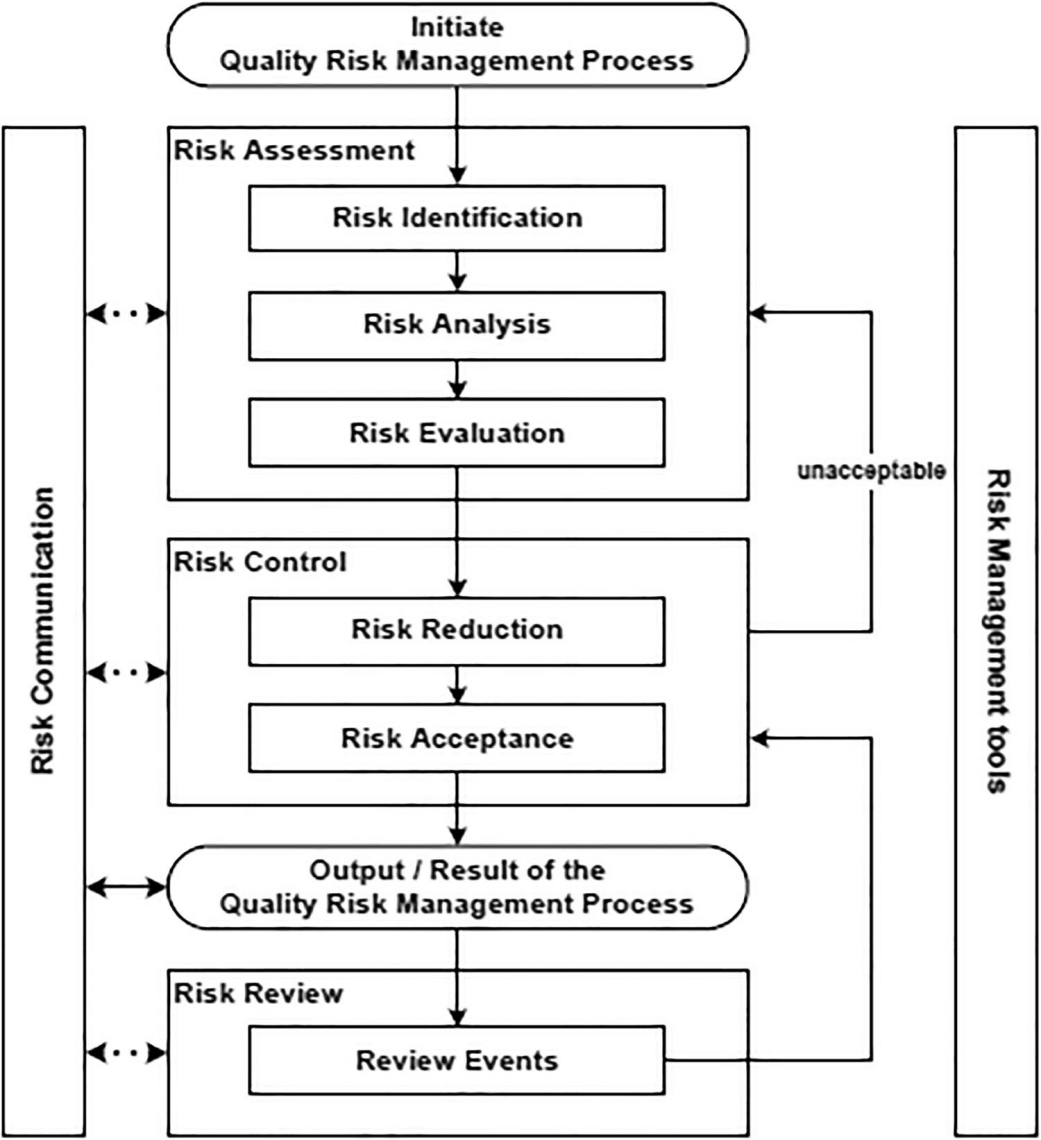
The key steps in QRM as described in ICH guideline Q9 on quality risk management [10]

QRM should be an integral element of the quality management system, and continuous efforts should be made to effectively manage the risks and optimise the efficiency of the preparation of radiopharmaceuticals. An approved procedure to describe the approach to risk management should be available. The structure and details of risk management procedures depend on many factors, such as site organisation, complexity and the variety of radiopharmaceutical preparation. The level of efforts placed in risk management should be commensurate with the level of risk. For small-scale “in-house” preparation of radiopharmaceuticals, a simplified QRM process may often be adequate. A schematic representation of a typical risk management process is given in Fig. 1.

### 1) Initiation of a risk assessment

To initiate and plan an assessment, the most important things to do are the following:

- Clearly define the aim.
- Select people with a thorough knowledge of the topic to be involved in the assessment process. It is useful to identify a leader.
- Define timelines, if applicable.
- Define the risk criteria and the acceptance levels (quantitative, qualitative, ranks of severity), which are needed for subsequent risk evaluation.

Defining the aim is very important. Thus, the basic question to be answered when initiating an assessment is “What is the precise purpose of this assessment?” instead of “Which outcome should be made?”

Following the above steps will result in an efficient process, minimising gaps in the assessment. The level of effort, resources, formality and documentation of the assessment should be commensurate with the level of risk, in line with ICH Q9 [10].

The initiation of an assessment may be triggered by several events, such as:

Deviations:

- A deviation (either planned or unplanned) from an established procedure
- An out of specification (OOS) result
- An adverse trend
- An unusual event or abnormal result, which does not necessarily represent a deviation but needs to be assessed and investigated.
  Changes:
- Substantial changes related to a process, product, method, equipment or material
- Introduction of a new radiopharmaceutical product, method, equipment and material
- Commissioning of a new radiopharmaceutical preparation laboratory
- Development of a new environmental monitoring program
  Standard processes:
- Requirements for storage of retention samples
- Vial labelling prior to radiopharmaceutical preparation
- Batch release before completion of quality control testing
- Approval of suppliers
- Requirements for chemical precursors for small-scale radiopharmaceutical preparation
- (Re)validation of facilities, equipment and processes

### 2) Risk assessment

Risk assessment is the “core” part of the risk management process and includes risk identification, analysis and evaluation.

The basic questions to be answered during the stages of the assessment process are the following:

- What might go wrong (identification of hazards)?
- What will happen, if it really goes wrong (impact on product quality, patient safety and efficacy)?
- How serious are the consequences (severity)?
- What is the probability of it going wrong (occurrence)?
- How easy is it to detect (detectability)?

#### Risk identification

The first step is to identify and list potential hazards related to the activity to be assessed. When they have been identified, they must be accurately described to ensure that their exact nature is defined. All available information should be used systematically. If the aim (defined during initiation of the risk assessment) is not clear, the wrong risk could be assessed, and ineffective corrective and preventative actions (CAPA) could be introduced. Risk (or hazard) identification provides the basis for the next stages of the assessment process. This stage addresses the “What might go wrong” question, including identifying the possible impact.

#### Risk analysis

The risk analysis consists of understanding and estimating the individual parts of the risk associated with each identified hazard. It is the process of ranking and linking the occurrence of the hazard and the severity of potential consequences, either qualitative or quantitative. Risk analysis addresses the questions “What is the probability (occurrence) of it going wrong”, and “What are the consequences, if it goes wrong, especially for the patient (severity)”. The ability to detect the consequences early enough (detectability) is also a factor of concern at this stage. For a robust output it is useful to differentiate clearly between these three factors: occurrence, severity and detectability.

#### Risk evaluation

At the risk evaluation stage, the significance of each identified risk is assessed by combining the outcome of the individual parts (occurrence, severity, detectability) based on the criteria defined during the risk initiation. Every combination of the individual parts (occurrence, severity, detectability) attached with a qualitative (“high”, “medium” or “low”) or quantitative (numeric value) description of risk should be considered. Risk evaluation not only leads to a qualitative or quantitative output of the risk assessment process, but also forms the basis for the following stage of risk control. The robustness of the data collected during the previous stages of the process will determine the quality of the output.

### Risk assessment tools

Numerous risk assessment tools are extensively described in ICH Q9 [10] and are beyond the scope of this guideline. A system based on qualitative or simple quantitative methods is generally considered to be adequate for small-scale radiopharmaceutical facilities. The choice of risk assessment tool should be made during initiation of the risk assessment. In this section, qualitative risk assessment, risk ranking and FMEA methods [11] are described.

#### Qualitative risk assessment

This is the simplest risk assessment method, whereby risks are classified as high, medium or low. The classification must be clearly defined before starting the risk analysis to ensure consistent results.

#### Risk ranking

This is the simplest quantitative assessment method, and it is applicable to assessment of simple processes. The result is a numeric output (risk priority number = RPN). The rating depends upon three factors:

i) Severity of the event while having no installed control measures (from no impact to severe impact)
ii) Occurrence (from highly unlikely to very probable)
iii) Detectability of the event (are controls and procedures in place to detect the error in good time)

By multiplying the above three factors, a quantitative description of the impact rating is obtained that helps to determine the level of investigation required and whether additional control measures need to be adopted.

A typical example is as follows:

**Table:** 

	Severity (S)	Description
Low	1	Expected to have little negative impact
Medium	2	Expected to have a medium negative impact
High	3	Expected to have a high negative impact

**Table:** 

	Occurrence (O)	Description
Low	1	Failure expected to happen less than once per year
Medium	2	Failure expected to happen once per year
High	3	Failure expected to happen more than once per year

**Table:** 

	Detectability (D)	Description
High	1	All failures are expected to be detected early enough
Medium	2	Some, but not all failures are expected to be detected early enough
Low	3	None of the failures are expected to be detected or are expected to be detected too late

By multiplying S × O × D, the RPN is calculated. Suitable action levels are defined based on the RPN, for example:

RPN > 12: High, unacceptable, action needed.
12 > RPN > 5: Medium, tolerable, further investigations to determine possible actions.
RPN < 5: Low, acceptable, no action needed.

#### FMEA

Failure mode and effects analysis (FMEA) is a more detailed assessment method which is used for complex processes [11]. It evaluates potential failure modes of a process and their likely effects on the product. The first step when conducting a FMEA is to break down a complex process into simpler steps; thus, this assessment tool relies on thorough product and process understanding. Subsequently, the potential failure modes (hazards and their impact) are identified and ranked, assigning an RPN to each potential risk based on previously defined scores for the severity, occurrence and detectability (see paragraph above). Once risks are identified and scored, risk reduction activities can be identified which change the numeric values of occurrence or detectability, resulting in a new RPN.

FMEA is a very useful tool to summarise possible failures, their causes, possible risk reducing activities and their impact on processes/products. It may potentially be applied to any equipment/facility and be used to analyse an operational step and its effect on product or process, to identify the parts of the process that are most in need of change. To perform a successful and useful FMEA, all the steps of the process must be identified with all the possible failure modes. Once the failure modes have been identified, all controls to avoid failure occurrence need to be listed, and the risk and likelihood of the failure evaluated. Examples of risk assessments using FMEA are given below in example 5 and in the literature [12].

### 3) Risk control

The outcome of risk control is a conscious decision regarding risk acceptance or reduction. The aim is to reduce all risks to an acceptable level, if possible and appropriate. The rationale for accepting the risk must be stated on a case-by-case basis:

- Which risks can be accepted because of their low level?
- Which risks can and need to be reduced or eliminated?
- Which risks cannot be reduced but are acceptable or require the cancellation of the assessed project?

In the first case (a), the risk is deemed acceptable, because its level is so low that specific actions are not necessary.

In the second case (b), the impact on product quality, efficacy or patient safety is so severe (unacceptable), that control measures must be implemented to reduce or eliminate the risk. These risk reducing activities can either decrease the occurrence (by validation and training activities) or increase the detectability (by additional quality controls or checks). It is not possible to change the severity for any given risk. The decision to reduce the risk may entail implementation of corrective and preventative actions, redesign of processes and writing or reviewing of documents.

In the third case (c), the risk is estimated unacceptable, but it is not possible to reduce it, or reduction is not considered feasible, so that the proposed control measures are rejected.

### 4) Risk communication

The extent of risk communication is related to the size and complexity of the production site and organisation but is of utmost importance. Risk communication should occur throughout the whole risk assessment process and should therefore be embedded in the risk management process. The importance of risk communication and who should be informed at which stage of the process should be clearly described in the associated procedure. While initiating a risk assessment process, it can be helpful to share the information for risk identification and evaluation with all relevant personnel in the organisation. At the very least, the completed risk assessment document should be adequately communicated to all relevant personnel.

### 5) Risk review

Once an assessment has been completed and corrective/preventative actions implemented when deemed appropriate, a review of the entire assessment process must be performed to assess its effectiveness. The risk review process should be embedded in the risk management process and should be clearly described in the risk management procedure. The outcome of the assessment should be reviewed:

- Regularly to consider new knowledge and experience
- After changes to assess their impact

## Practical examples

Examples of risk assessments associated with radiopharmaceuticals are presented below, covering several different situations and factors where a risk assessment may be appropriate.

### Risk assessment required for a standard process

*Example 1: Qualitative output—batch release before completion of quality control testing for PET radiopharmaceuticals*

Due to the short half-life of their radionuclides, PET radiopharmaceuticals may be released before the results of the test for sterility, radionuclidic purity test and environmental monitoring are available. This can only be accepted if an assessment is carried out to make a rationale decision about the associated risks. This simple assessment is carried out qualitatively in the following example:

**Table:** 

Introduction
Annex 3 “Manufacture of Radiopharmaceuticals” outlines the requirements for manufacture of radiopharmaceuticals. Adherence to this annex is associated with the following premise (as stated in the annex): *Due to short shelf-life of their radionuclides, some radiopharmaceuticals may be released before completion of all quality control tests. In this case, the exact and detailed description of the whole release procedure including the responsibilities of the involved personnel and the continuous assessment of the effectiveness of the quality assurance system is essential*
Aim
The aim is to decide if and under which conditions it can be accepted to release the product before completion of all QC tests (here: test for sterility and radionuclidic purity)
Risk identification
The following hazards can occur, when the product is released before completion of the test for sterility and radionuclidic purity test: • Sepsis in the case of microbial contamination • Extra radiation dose due to radionuclidic impurities
Risk analysis
The risk of releasing radiopharmaceuticals before completion of all quality control tests is high as this could lead to a negative impact on the patient. This risk is mitigated by the fact that only small amounts of the product are administered to patients, all processes are validated, an adequate quality management system is in place and personnel involved in production, quality control and release of radiopharmaceuticals are appropriately trained in specific radiopharmaceutical aspects of the quality management system. All manufacturing steps take place in self-contained facilities dedicated to radiopharmaceuticals, accessible only by authorised personnel. Measures are established and implemented to prevent cross-contamination. Preventative maintenance, calibration and qualification programmes ensure that all facilities and equipment used in the manufacture of radiopharmaceutical are suitable and qualified. The facilities are routinely monitored so that the appropriate level of environmental cleanliness is maintained. The starting materials, packaging materials and critical process aids are purchased from approved suppliers. All documents related to the manufacture of radiopharmaceuticals are prepared, reviewed, approved and distributed according to written procedures. A written procedure detailing the assessment of production and analytical data is followed before the batch is released
Risk evaluation and control
With the adopted risk reducing activities, the risks are considered to be tolerable. All risks are accepted, on the condition that the described risk reducing activities are conducted

*Example 2: Quantitative output—requirements for chemical precursors for small-scale radiopharmaceutical preparation*

When using a chemical precursor for a radiopharmaceutical preparation, there is no need for risk assessment if the chemical precursor complies with a monograph in the European Pharmacopoeia (in this case, the risk assessment was done by people with acknowledged expertise). When there is no individual monograph in the European Pharmacopoeia, then the general monograph “Chemical precursors for radiopharmaceutical preparations” (2902) is applicable. Points which do not comply with the monograph should be risk assessed on a case-by-case basis. This simple assessment is carried out quantitatively in the following example:

**Table:** 

Introduction:
The precursor used for the preparation of PET radiopharmaceutical XXX is fully compliant with Ph. Eur. monograph 2902 except for the requirements for microbial contamination and bacterial endotoxins
Aim
The aim is to decide if and under which conditions the chemical precursor for a radiopharmaceutical preparation can be used, although there is no data on microbiological and endotoxin contamination
Risk identification:
The product may not be sterile and contain excessive levels of bacterial endotoxins
Risk analysis:
Severity: (3)
The risk of using a product that does not meet the sterility and endotoxin requirements is high as this could be detrimental to the patients health
Detectability: (1)
Controls and procedures are in place which will detect any microbial or endotoxin contamination of the precursor. A bioburden test of the radiopharmaceutical product (without terminal sterile filtration) is performed for every new batch of precursor as part of the incoming goods approval process. Endotoxin testing is performed on the radiopharmaceutical product before release
Occurrence: (1)
This failure is expected to happen less than once a year. The precursor is provided with a certificate of analysis which certifies the chemical purity which is specified at > 97%. YYY is a well trusted supplier and has been audited regularly and found to have a well-established quality management system. Each batch of product is sterile filtered, and each filter is tested for integrity after use. Each batch of product is tested for endotoxins prior to release
RPN = 3 × 1x1 = 3 Low, acceptable
Risk evaluation and control
The risk is considered acceptable with the installed reducing activities including at least a bioburden test of the product with every new batch of precursor and an endotoxin test of the radiopharmaceutical product before release

### Risk assessment triggered by deviations

In case of a deviation, a risk assessment may be needed to assess the impact that the event can have on the process/product and ultimately on patients, to identify the root cause and to identify corrective/preventative actions when necessary. The outcome of the assessment may be to accept the deviation if it is considered an isolated event and its impact is of no concern.

In the case of minor, but repeated deviations, the outcome of the assessment may still be to accept the deviation, but corrective/preventative actions may be considered necessary to prevent reoccurrence. For instance, if the reactor of a radiosynthesis module is not performing as expected and validated and it takes longer to heat up, it is possible that it will reach the point where it fails to reach the required temperature and the radiosynthesis fails to produce a radiopharmaceutical that meets the required specifications. Finally, if the deviation is considered to have a medium/major impact, the product may be rejected, and a thorough investigation may be needed to understand the root cause and resolve the problem.

A deviation procedure must be available within the quality management system, which describes how to identify and evaluate deviations, how to document them (i.e. a deviation form), the approval flow and the responsibilities of personnel involved in the investigation. The classification of the deviations (i.e. minor/medium/major, planned/unplanned) must also be well described. The deviation procedure should also describe how to perform a root cause analysis and how to identify corrective and preventative actions when necessary. The most used root cause analysis tool is the “5 whys”. When using the 5 whys tool, the aim is to identify the most probable root cause(s) by starting with a defined problem statement and asking “why did this happen” until you cannot ask the question anymore, as you have reached the final reason that caused the event. The number “5” is not restrictive; it can take more questions or even less questions to identify the most probable root cause.

In the following two examples, a deviation form is used to record the deviation, the assessment, the root cause analysis (using the 5 whys tool) and any corrective actions, according to an approved deviations procedure.

*Example 3: Risk assessment within a deviation form due to a minor deviation* 
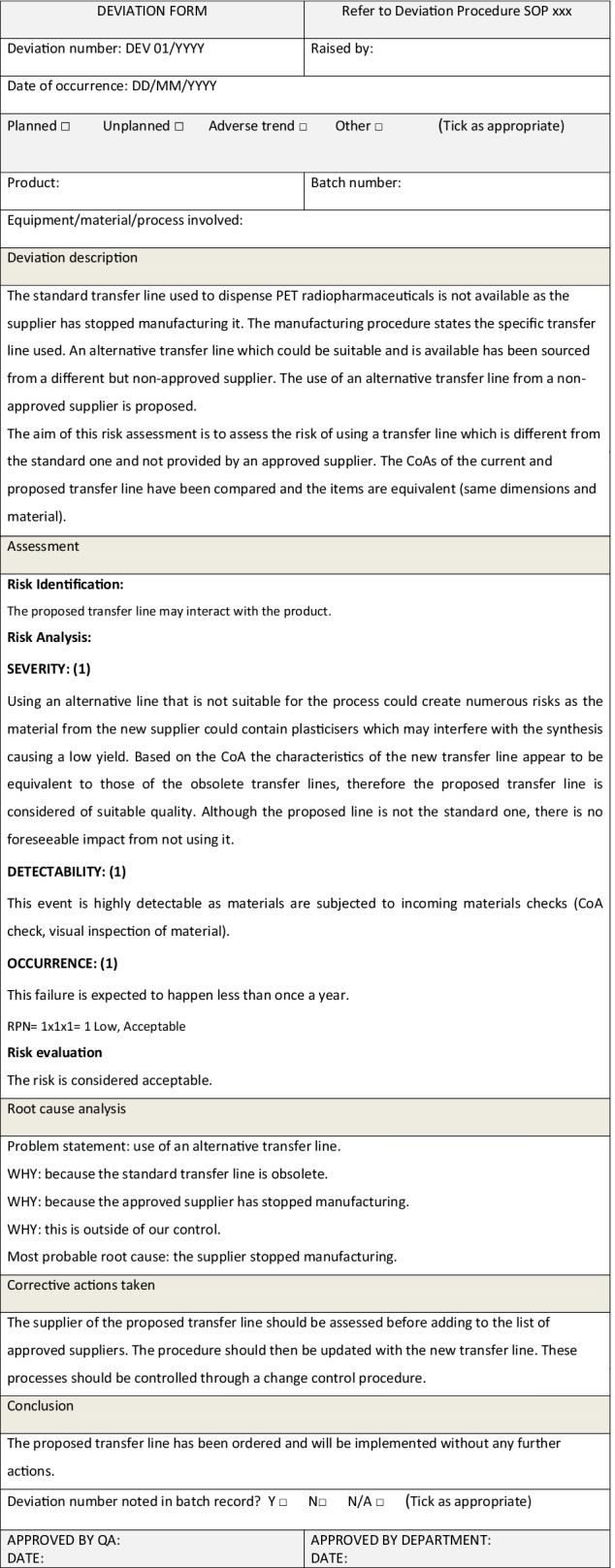


*Example 4: Risk assessment within a deviation form due to a major deviation* 
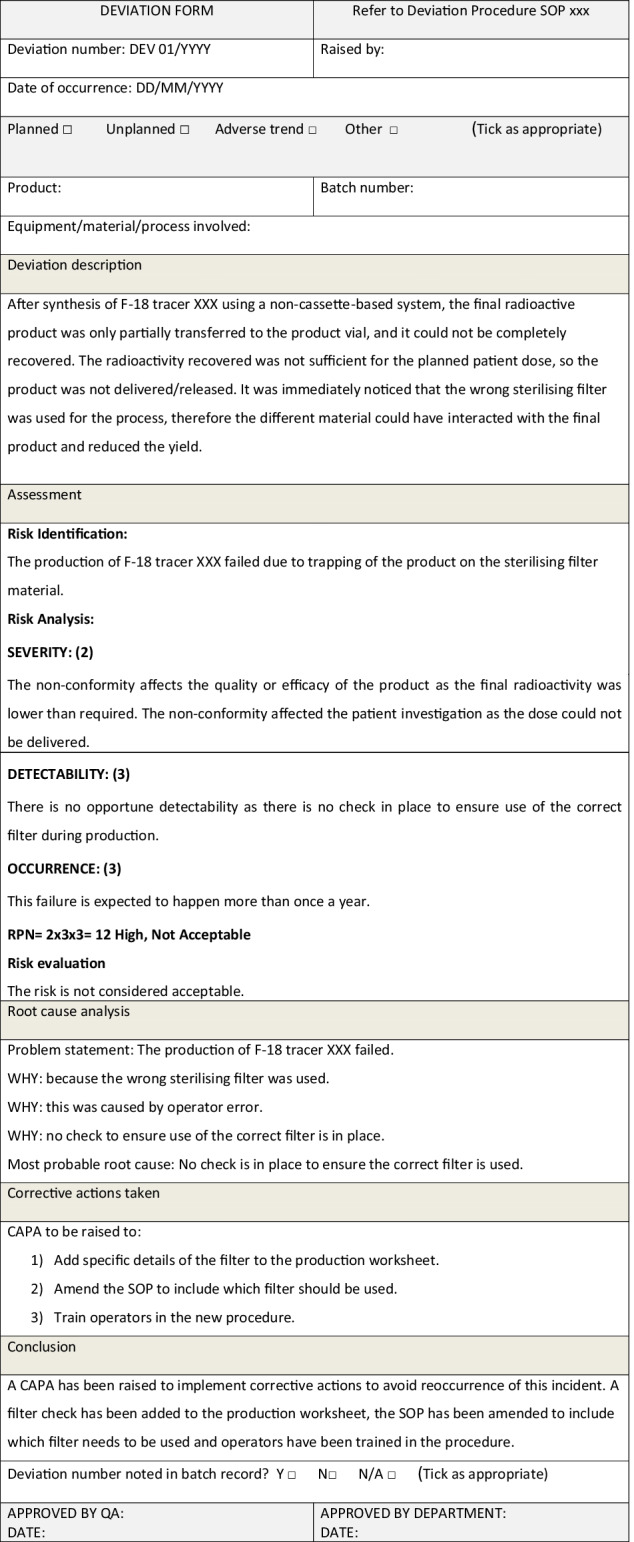


*Example 5: Risk assessment within a change control form: introduction of a new product synthesised using a cassette system* 
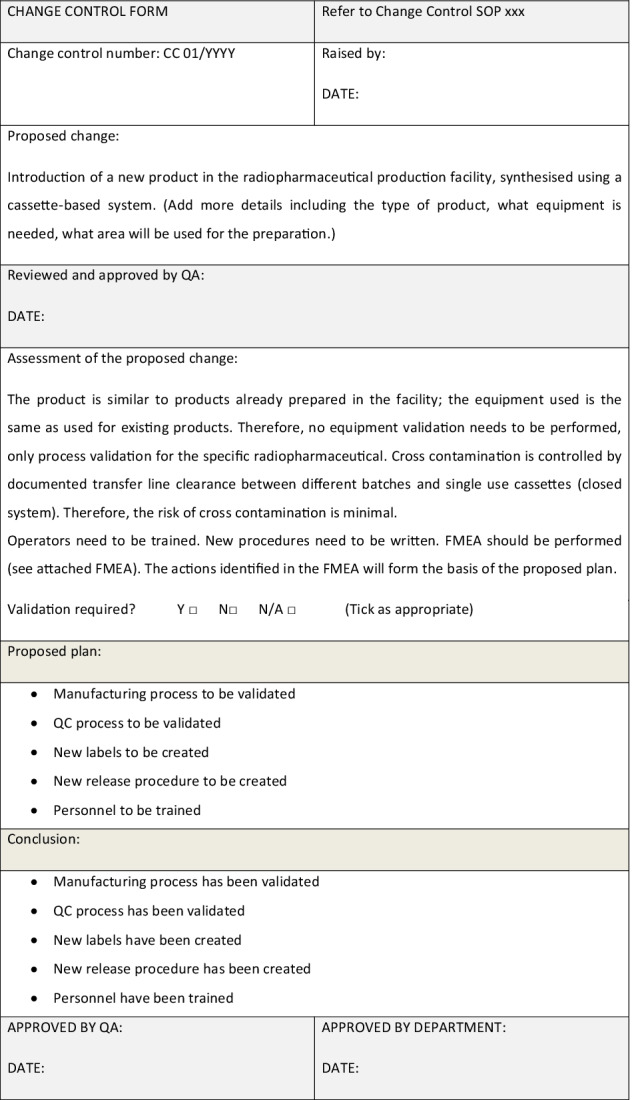


### Risk assessment triggered by changes

When introducing a new radiopharmaceutical product, method, equipment or material or in the case of a substantial change, a risk assessment may be needed to assess the impact on the patients’ safety and to assess and identify the level of validation required [13]. Commissioning of a new radiopharmaceutical preparation laboratory should also use change management, and a risk assessment should be performed to assess and identify the level of validation required.

A change control procedure should be available to ensure changes are implemented in a controlled manner. If a change is not performed using change management, it is possible that the impact on the validation status of the equipment/process has not been considered, and therefore, the equipment/process is no longer compliant. For example, if a transfer line is changed without an assessment and a material that is not compatible with the radiopharmaceutical is used, the final product could stick to the new material, and the radioactivity recovered in the final product vial could be insufficient.

The change control procedure should specify the workflow and the responsibilities involved. The proposed change should be reviewed and approved by relevant personnel (e.g. process/equipment owner and QA). The proposed change should be risk-assessed by the relevant personnel (e.g. QC or production managers), to ensure it is sufficiently detailed to make an informed decision. Consequently, the change could be either rejected (e.g., because it is not necessary, not sufficiently detailed or holds unacceptable risks) or approved. If the change is approved, a plan should be created and approved by suitable personnel including QA. At this stage, the change is ready to be implemented in a controlled way. Example 5 below includes an example of change control form. After implementation, the change and the associated risk assessment should be reviewed to ensure the change has been implemented correctly.

In the following example, a new radiopharmaceutical product is introduced in a facility. This change is introduced via change management, using a change control form. As it is a complex change with several different factors to be considered, a FMEA is used to perform the risk assessment and to identify any required actions.

If a new radiosynthesis module, new materials and new QC equipment and methods are used, the risks identified in the assessments will usually be considerable, and thus, a full validation will be required (IQ, OQ, PQ). If the new radiopharmaceutical is prepared using a fully validated radiosynthesis module already in use, fully validated QC equipment but different analytical methods, the risks identified in the assessment will usually be lower, and a lower level of validation will be required.

Attached FMEA:

**Table:** 

Process	Potential failure mode	Potential failure effects	Severity	Current controls	Occurrence	Detectability	RPN	Actions for the proposed plan, which complete the current controls
Label production	Labels contain incorrect information	Product incorrectly labelled	Medium (2)	Current label process to be used. The labels are double checked before approval for use and before use	Very low (1)	High (1)	2	New labels needed
Manufacturing	Production fails	Radiopharmaceutical batch is not ready for the patients	High (3)	Current validation policy will ensure that processes are validated	Very low (1)	High (1)	3	Validation of the new manufacturing process
QC	QC fails	Radiopharmaceutical batch may not be released	High (3)	Current validation policy will ensure reliability of the process. The analytical method will be validated. The equipment is already validated	Very low (1)	High (1)	3	Validation of new analytical methods
Release	Product is not released	Radiopharmaceutical is not available for the patients	High (3)	The process will be performed according to approved procedures, by trained operators	Very low (1)	High (1)	3	New procedures needed. Training to be performed

## Data Availability

Not applicable.

## Appendix

**Appendix 1:** Abbreviations

EANM	European Association of Nuclear Medicine
cGRPP	Current Good Radiopharmacy Practice
QMS	Quality management system
SOP	Standard operating procedure
CAPA	Corrective actions and preventative actions
FMEA	Failure mode and effects analysis
ISO	International Organization for Standardization
OOS	Out of specification
OOT	Out of trend
Ph. Eur	European Pharmacopoeia
QC	Quality control
QRM	Quality risk management
RP	Radiopharmaceutical
RPN	Risk priority number

**Appendix 2:** Glossary

Abnormal result	Results that are still within specification but are unexpected, unusual
Adverse trend	An identified trend that indicates a degradation in performance
CAPA	Corrective actions and preventative actions. A CAPA process is a formal way to implement appropriate corrective and preventative actions with the aim of eliminating causes of non-conformity such as deviations, OOTs, deficiencies from audits and other complaints. Prior to initiation of a CAPA process, non-conformities must be investigated systemically to determine the root cause. CAPA methodology should result in product and process improvements and enhanced product and process understanding
Change management	A systematic approach to proposing, evaluating, approving, implementing and reviewing changes that ensures that changes to methods, equipment and materials are properly documented and assessed before implementation
Corrective actions	Actions taken to eliminate the cause of a detected non-conformity to prevent recurrence
Detection	The process of identifying a failure mode
Deviation	A non-conformity with respect to approved procedures or established standards or specifications. A deviation can be planned or unplanned. A planned deviation is pre-approved and covers a specified period or number of batches
Failure mode	Different ways that a process or sub-process can fail to yield the anticipated result
Failure mode and effects analysis	The process of reviewing as many components, assemblies, and subsystems as possible to identify potential failure modes in a system and their causes and effects
Good radiopharmacy practice	Good radiopharmacy practice is described in the “Guidelines on current Good Radiopharmacy Practice (cGRPP)” by the Radiopharmacy Committee of the EANM﻿ [14]
Occurrence	Probability that an event may happen within a given timeframe
Preventative actions	Actions taken to eliminate the cause of a potential non-conformity to prevent its occurrence
Product life cycle	All stages in the life of the product, from development through its clinical use
Quality risk management (QRM)	A systematic process for the assessment, control, communication, and review of risks to the quality of the pharmaceutical product throughout the product life cycle. Refer to Fig. 1 for key steps in QRM
Risk	Combination of the occurrence and severity of a hazard (potential source of harm) or non-conformity
Risk assessment	Risk assessment is the initial step towards collecting information to support a risk decision. It consists of three main stages
Risk identification	The first stage of the risk assessment process, during which hazards are identified. All available information should be used systematically
Risk analysis	The second stage of the risk assessment process, during which the risk associated with the identified hazard or non-conformity is estimated
Risk evaluation	The third stage of the risk assessment process, during which the significance of the risk is determined by comparison of the answers provided during the risk identification and risk analysis stage with given risk criteria (e.g. quantitative scale or qualitative levels such as high, medium and low)
Risk control	This step follows the risk assessment, and the output is a decision regarding risk acceptance or reduction. Which risks can be accepted because of their low level, which risks can and need to be reduced/eliminated and which risks cannot be reduced but are accepted without reduction given an acceptable risk–benefit ratio
Risk reduction	Actions taken to decrease risk to a lower level. This can be achieved by reducing the occurrence or enhancing the detectability
Risk acceptance	An active decision to accept an identified risk
Risk communication	The act of sharing information and outcomes of a risk assessment
Risk review	Review or monitoring of output or results of a risk assessment considering (if appropriate) new knowledge and experience
Risk priority number (RPN)	A numeric, quantitative assignment of the level of risk associated with a process or steps in a process. Each failure mode is assigned a numeric score that quantifies the likelihood of occurrence, likelihood of detection and severity of impact. The product of these three scores is the RPN for that failure mode. RPN = severity rating × occurrence rating × detection rating
Root cause analysis	A systematic process for identifying root causes of problems conformities or other adverse events
Validation	The documented actions of confirming that any procedure, process, equipment, material, activity or system leads to the expected results

## Declarations

### Liability statement

This guideline summarises the views of the EANM Radiopharmacy Committee. It reflects recommendations for which the EANM cannot be held responsible. The recommendations should be taken into the context of good practice of nuclear medicine and do not substitute for national and international legal or regulatory provisions Tables 1 and 2.
